# Early outcomes of patella resurfacing in total knee arthroplasty: A report from the Australian Orthopaedic Association National Joint Replacement Registry

**DOI:** 10.3109/17453670903413145

**Published:** 2010-03-31

**Authors:** Warren J Clements, Lisa Miller, Sarah L Whitehouse, Stephen E Graves, Philip Ryan, Ross W Crawford

**Affiliations:** ^1^Orthopaedic Research Unit, Prince Charles Hospital, Brisbane; ^2^Data Management and Analysis Centre, Discipline of Public Health, University of Adelaide, Adelaide; ^3^Institute of Health and Biomedical Innovation, Queensland University of Technology, Brisbane; ^4^Australian Orthopaedic Association National Joint Replacement Registry, AdelaideAustralia

## Abstract

**Background:**

Patella resurfacing in total knee arthroplasty is a contentious issue. The literature suggests that resurfacing of the patella is based on surgeon preference, and little is known about the role and timing of resurfacing and how this affects outcomes.

**Methods:**

We analyzed 134,799 total knee arthroplasties using data from the Australian Orthopaedic Association National Joint Replacement Registry. Hazards ratios (HRs) were used to compare rates of early revision between patella resurfacing at the primary procedure (the resurfacing group, R) and primary arthroplasty without resurfacing (no-resurfacing group, NR). We also analyzed the outcomes of NR that were revised for isolated patella addition.

**Results:**

At 5 years, the R group showed a lower revision rate than the NR group: cumulative per cent revision (CPR) 3.1% and 4.0%, respectively (HR = 0.75, p < 0.001). Revisions for patellofemoral pain were more common in the NR group (17%) than in the R group (1%), and “patella only” revisions were more common in the NR group (29%) than in the R group (6%). Non-resurfaced knees revised for isolated patella addition had a higher revision rate than patella resurfacing at the primary procedure, with a 4-year CPR of 15% and 2.8%, respectively (HR = 4.1, p < 0.001).

**Interpretation:**

Rates of early revision of primary total knees were higher when the patella was not resurfaced, and suggest that surgeons may be inclined to resurface later if there is patellofemoral pain. However, 15% of non-resurfaced knees revised for patella addition are re-revised by 4 years. Our results suggest an early beneficial outcome for patella resurfacing at primary arthroplasty based on revision rates up to 5 years.

## Introduction

Early knee arthroplasty designs without patella resurfacing were associated with higher rates of patello-femoral problems including anterior knee pain, patella subluxation, and patella erosion (Insall et al. 1976). Aglietti et al. (1975) described the design of a patella component based on the area of articulation and loading in the cadaveric patellofemoral joint.

Resurfacing of the patella at primary surgery has always been a contentious issue, and recent studies remain conflicting. Boyd et al. (1993) suggested that replacement of the patella in patients with osteoarthritis and rheumatoid arthritis prevents early revision. This was supported by Burnett and Bourne (2003) who analyzed results from 5 randomized controlled trials (Schroeder-Boersch et al. 1988, Bourne et al. 1995, Feller et al. 1996, Barrack et al. 2001, Wood et al. 2002), and showed that of 451 knees having total arthroplasty, 11% without patella resurfacing required revision as compared to 5% of knees with patella resurfacing. Anterior knee pain was the most common complication in the non-resurfaced groups. These results have been supported by other literature suggesting resurfacing of the patella leads to lower rates of revision (Forster 2004, Pakos et al. 2005, O'Shea et al. 2006, Garneti et al. 2008) or increased patient satisfaction (Schroeder-Boersch et al. 1998, Mayman et al. 2003, Waters and Bentley 2003, Burnett et al. 2004, Gildone et al. 2005, Parvizi et al. 2005, Berti et al. 2006, van Hemert et al. 2009). Despite promising results, other studies have suggested that resurfacing of the patella does not change rates of revision, patient satisfaction, or clinical outcome (Grace and Sim 1988, Healy et al. 1995, Robertsson et al. 2000, Wood et al. 2002, 2005, Burnett et al. 2004, 2007, Campbell et al. 2006, Myles et al. 2006, Oztürk et al. 2006, Smith et al. 2006, 2008, Epinette and Manley 2008). Most studies to date have been under-powered and the role of patella resurfacing in total knee arthroplasty is not clearly defined.

Whether to resurface the patella at primary surgery or as a subsequent reoperation is also unclear. Surgeons commonly believe that resurfacing as a secondary procedure is as beneficial as resurfacing at the initial operation. Surgeons who choose not to resurface the patella in the primary arthroplasty may consider it easy to resurface the patella later if the patient experiences complications such as patellofemoral pain. However, Khatod et al. (2004) and Muoneke et al. (2003) reported that only half of these patients will have satisfactory results. To date, there has been no literature suggesting that the revision rate is the same when resurfacing at primary surgery or at revision, in the context of total knee arthroplasty.

Much of the literature concerning patella resurfacing in total knee arthroplasty states outcome for osteoarthritis alone (Feller et al. 1996, Burnett et al. 2004, Campbell et al. 2006). Boyd et al. (1993) suggested a beneficial outcome for resurfacing irrespective of the diagnosis. Despite this, there has been no specific comparison of the outcomes of patella resurfacing by diagnosis; thus, the outcome of resurfacing the patella for different diagnoses remains uncertain.

We used data from the Australian Orthopaedic Association (AOA) National Joint Replacement Registry (NJRR) to investigate the use of patella resurfacing in total knee arthroplasty.

## Patients and methods

Ethics approval was obtained from the Prince Charles Hospital Human Research and Ethics Committee prior to requesting data.

The purpose of the Commonwealth Government funded AOA NJRR is to improve the quality of care for patients undergoing joint replacement surgery. Similar registries exist in other countries, including the Swedish Knee Arthroplasty Register, which has been in operation since 1976 (Knutson et al. 1994). The AOA NJRR commenced data collection in 1999 and has collected full national data since mid-2002, with a greater than 97% capture rate. All 289 hospitals (public and private) currently undertaking joint replacement surgery in Australia provide information to the Registry. The 2007 annual report analyzed 172,349 knee procedures performed between September 1, 1999 and December 31, 2006, of which 134,799 were total knee arthroplasties. Data obtained at the time of surgery include patient details, hospital, type of procedure, joint replaced, side (left or right), diagnosis, and details of all components used. Although some identifying information including names are collected, no patient, surgeon, or hospital is identified in any data released by the AOA NJRR (Graves et al. 2004).

The main outcome reported by the registry is time to first revision. As the registry is still in its infancy, data reflect early rates of revision, although the very substantial number of procedures collected make it a valuable source of information to compare outcomes (Graves et al. 2004, Robertsson 2007).

### Statistics

The cumulative per cent revision (CPR) of primary total knee arthroplasty at each of the first 5 years following implant was estimated using the Kaplan-Meier method. Our main interest was to compare revision rates between resurfaced patellas at primary arthroplasty (the resurfacing group, R) and non-resurfaced patellas at primary arthroplasty (the no-resurfacing group, NR). Of secondary interest was the outcome of revision procedures after the primary arthroplasty (R and NR) where the components inserted at the time of revision surgery were the “patella only” or the “patella and insert” (thus excluding “insert only”). Finally, revision rates for R and NR were compared between primary diagnosis of osteoarthritis and all other diagnoses. Here “other diagnosis” refers for example to rheumatoid arthritis, other inflammatory arthritis, avascular necrosis, tumors, and chondrocalcinosis.

Unadjusted CPR values are reported with 95% confidence interval (CI). Adjustment for age and sex was made, where appropriate, when comparing revisions over the entire period, using either log-rank tests or hazards ratios from proportional hazards models as appropriate. All tests were two-tailed at the 5% level of significance.

Descriptive analyses including primary diagnosis, reasons for revision, and type of revision are also reported. Type of revision was categorized into major (involving femoral and/or tibial components) or minor (not involving femoral and/or tibial components).

Analyses were performed using SAS software version 9.1 (SAS Institute Inc., Cary, NC).

## Results

Of the 134,799 primary total knee arthroplasties reported in the 2007 annual report, 57,359 (43%) involved patella resurfacing. In the R group, 93% were cemented.

**Figure 1.**
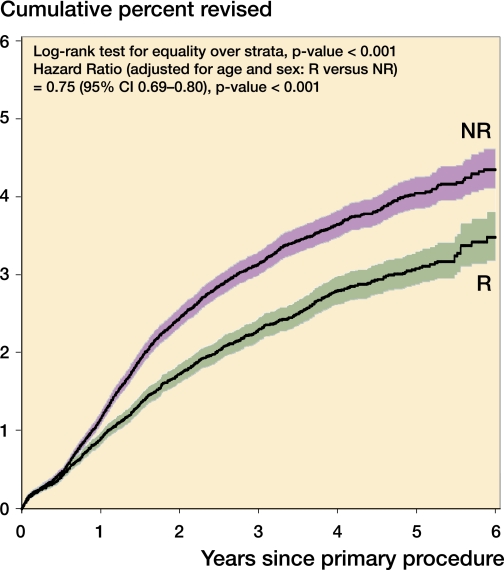
Cumulative percent revision (CPR) of primary total knee arthroplasty by patella resurfacing.

Primary total knee arthroplasty in the R group had a lower revision rate than in the NR group (adjusted HR = 0.75, CI: 0.69–0.80; p < 0.001) (Figure 1). At 5 years, the CPR of total knee procedures for the R group was 3.1% as compared to 4.0% for the NR group (Table 1).

**Table 1.** Annual cumulative percent revision (CPR) of primary total knee arthroplasty by patella resurfacing

Patella resurfacing at primary operation	Cumulative percent revision after				
	1 year	2 years	3 years	4 years	5 years
Non-resurfaced patella (NR)	1.2 (1.1–1.2)	2.5 (2.3–2.6)	3.1 (3.0–3.3)	3.6 (3.5–3.8)	4.0 (3.9–4.3)
Resurfaced patella (R)	0.9 (0.8–1.0)	1.7 (1.6–1.9)	2.3 (2.1–2.4)	2.8 (2.6–3.0)	3.1 (2.9,–3.3)

The most common reasons for revision in both groups were loosening and infection. However, in the R group, loosening (36%) and infection (27%) were more common than in the NR group (29% loosening, 19% infection) (Table 2). Conversely, in the NR group, patellofemoral pain (17%) and knee pain (13%) were more common reasons for revision than in the R group (1.1% patellofemoral pain, 7.0% knee pain) (Table 2).

**Table 2.** Reason for revision of primary total knee arthroplasty by patella resurfacing

Reason for revision **^a^**	Non-resurfaced patella (NR)		Resurfaced patella (R)		Total	
	N	%	N	%	N	%
Loosening	606	29	421	36	1,027	31
Infection	389	19	323	28	712	22
Patellofemoral pain	361	17	13	1.1	374	11
Pain	270	13	82	7.0	352	11
Instability	97	4.6	59	5.0	156	4.8
Arthrofibrosis	78	3.7	55	4.7	133	4.1
Fracture	37	1.8	45	3.8	82	2.5
Malalignment	38	1.8	27	2.3	65	2.0
Dislocation	14	0.7	10	0.9	24	0.7
Patella maltracking	15	0.7	7	0.6	22	0.7
Wear, patella	19	0.9	1	0.1	20	0.6
Bearing/dislocation	10	0.5	9	0.8	19	0.6
Other	163	7.8	119	10	282	8.6
Total	2,097	100	1,171	100	3,268	100
^a^ Some patients had multiple diagnoses.						

There were 1,092 revisions of knees in the R group, of which 65 were for isolated patella revision (6%) while 626 were for tibia and/or femoral components (57%). Major revisions in the R group constituted 1.2% of all procedures with patella resurfacing. There were 1,979 revisions of knees in the NR group, of which 566 were for isolated patella addition (29%) and 762 for tibia and/or femoral components (39%). Major revisions in the NR group constituted 1.1% of all procedures without patella resurfacing. Patients in the R group showed a higher proportion of major revisions (p < 0.001) while those in the NR group showed a higher proportion of minor revisions (p < 0.001).

**Figure 2.**
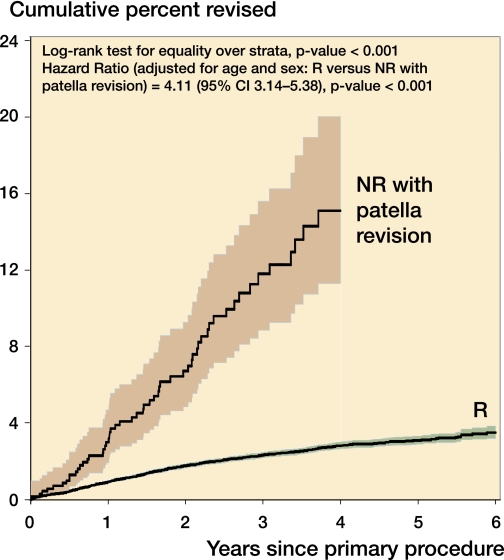
Cumulative percent revision (CPR) comparing patella resurfacing at primary surgery with non-resurfacing at primary surgery revised for patella resurfacing

There was a higher CPR in revisions for patella addition in the NR group than in the R group (adjusted HR = 4.1, CI: 3.1–5.4, p < 0.001). At 4 years, the CPR for the R group was 2.8% as compared to 15% for the NR group revised for patella addition (Figure 2), most (74%) of these being for patellofemoral pain.

Diagnosis at primary arthroplasty was similar for both groups, with 96% of the R group having osteoarthritis as compared to 97% of the NR group. For the NR group, the 5-year CPR for the diagnosis of osteoarthritis was 4.9% and for other diagnoses it was 4.0% (adjusted HR = 1.1, CI: 0.8–1.2; p = 0.7). For the R group, the 5-year CPR for the diagnosis of osteoarthritis was 3.1% and for other diagnoses it was 2.6% (adjusted HR = 1.7, CI: 1.2–2.4; p = 0.003) (Table 4). Other covariates including age at primary procedure, sex, and mean time to revision had no influence on revision rate between the diagnosis groups (data not shown).

**Table 3.** Type of revision for primary total knee arthroplasty comparing the use of patella resurfacing and no resulfacing

Type of revision	Non-resurfaced patella (NR)		Resurfaced patella (R)		Total	
	N	%	N	%	N	%
Tibial and femoral	336	17	340	31	676	22
Patella only	566	29	65	6.0	631	21
Insert only	323	16	277	25	600	20
Tibial only	184	9.3	172	16	356	12
Femoral only	242	12	114	10	356	12
Insert and patella	214	11	19	1.7	233	7.6
Cement spacer	73	3.7	71	6.5	144	4.7
Other minor components	20	1.0	15	1.4	35	1.1
Removal of prosthesis	19	1.0	15	1.4	34	1.1
Fusion nail	1	0.1	2	0.2	3	0.1
Reinsertion of components	1	0.1	2	0.2	3	0.1
Total	1,979	100	1,092	100	3,071	100

**Table 4.** Annual cumulative percent revision (CPR) of primary total knee arthroplasty by patella resurfacing and primary diagnosis

		Cumulative percent revision after				
Patella usage	Primary diagnosis	1 year	2 years	3 years	4 years	5 years
Non-resurfaced patella	Osteoarthritis	1.2 (1.1–1.2)	2.4 (2.3–2.6)	3.1 (3.0–3.3)	3.6 (3.4–3.8)	4.0 (3.8–4.2)
Non-resurfaced patella	Other diagnosis	1.2 (0.8–1.8)	2.9 (2.2–3.8)	3.5 (2.7–4.5)	4.5 (3.5– 5.7)	4.9 (3.8–6.3)
Resurfaced patella	Osteoarthritis	0.9 (0.8–1.0)	1.8 (1.6–1.9)	2.3 (2.2–2.5)	2.8 (2.6–3.0)	3.1 (2.9–3.3)
Resurfaced patella	Other diagnosis	0.5 (0.3–0.9)	1.1 (0.7–1.7)	1.7 (1.2–2.5)	2.2 (1.5–3.2)	2.6 (1.8–3.9)

## Discussion

We used registry data obtained from the AOA NJRR to compare rates of early revision in patients with and without patella resurfacing. We have addressed the pitfall of many previous studies, which lacked sufficient power to show any difference between rates of revision. The strengths of our study include a large sample size, the use of data reflecting current practice, and incorporation of data from many centers both public and private. The limitations of the study are that the only outcome was the rate of revision, while other measures such as Knee Society scores, patient satisfaction, and extensor function were not available. There are also many implant types with different individual variations in design, and as such any discrepancy in outcomes of patella resurfacing from each individual design was not adjusted for. Data from the registry reflect early revisions up to approximately 5 years.

In the recent literature, it has been proposed that revision rates are lower in patients who have received patella resurfacing in total knee arthroplasty (Lindstrand et al. 2001, Forster 2004, Pakos et al. 2005, O'Shea et al. 2006, Garneti et al. 2008). This was confirmed in our study, as we found that the R group had a lower revision rate than the NR group, with a hazards ratio of 0.75 (p < 0.001).

We found that patients in the NR group were more likely to be revised for patellofemoral pain, and more likely to be revised with isolated patella addition. Surgeons may be more inclined to revise a non-resurfaced knee by secondary patella addition if the patient presents later with knee pain, given that option is still available. While the etiology of anterior knee pain following total knee arthroplasty is not proven, the interplay of forces on the patellofemoral joint is thought to be the culprit (Mochizuki and Schurman 1979). However, in patients for whom there are other causes for anterior knee pain (e.g. subclinical infection, component rotation, anatomical abnormality, patella maltracking), a tendency to offer patella addition may not correct the cause of pain or could lead to incorrect treatment and the need for further major re-revision. Sharkey et al. (2002) discussed the concept of failing total knee arthroplasties and pointed out that early failure might be due to a number of mechanisms. In approximately 8% of patients who are generally dissatisfied with their knee arthroplasty (Robertsson et al. 2000), the ability to offer a minor revision in the absence of a diagnosis may further increase the rate of early revision.

If a patella is inserted at revision surgery we showed a higher rate of re-revisions than revisions of a knee where the patella was replaced at the primary operation (Figure 2). The 4-year cumulative per cent revision for NR with patella addition was 15%, with most revisions for loosening and infection requiring major re-revision. These results suggest patella resurfacing is more effective in terms of early revision when performed at the primary arthroplasty rather than at the first revision. We support reports suggesting that isolated patella addition in the non-resurfaced knee is associated with poor clinical outcomes and high rates of re-revision (Berry and Rand 1993, Leopold et al. 2003, Muoneke et al. 2003, Khatod et al. 2004), although this is the first study to compare primary and revision outcomes of patella resurfacing.

We identified a statistically significantly higher proportion of major revisions than minor revisions in the R group, and minor revisions compared to major in NR, with a higher proportion of revisions for loosening and infection in the R group than in the NR group. These rates support early data from the Swedish Knee Arthroplasty Register (Robertsson et al. 2001). Major revisions tended to occur later in the R group than major revisions in the NR group. Although these results were significant, the difference is probably related to a tendency to offer minor revisions to patients with no resurfacing as mentioned previously, particularly patients who are generally dissatisfied. A relatively simple patella addition is not available for patients with resurfacing, and this being the case surgeons may be inclined to wait and operate later with a major revision. This could account for both the lower proportion of major revisions and the lower proportion of loosening and infection (rather than patellofemoral pain) in the NR. As the registry does not collect data on operation time or the use of other infection control measures, we are unable to report on whether there may be a link between operation time and infection rates for resurfacing of the patella at the primary procedure; however, this is clearly a subject for further research.

It remains to be seen whether early outcome will be similar to long-term outcome. Currently, data are only available for the last 5 years, and the possibility of patella resurfacing having an adverse long-term effect on major components cannot be excluded without continuous data collection and further analysis. In addition, the integrity of the patella and its implanted button is also a long-term issue that remains to be investigated, and patella-related outcomes should be explored when further data become available. Given the close relationship between patella-related outcomes from the Australian and Swedish registries, it is possible that Australian long-term outcome may reflect Swedish long-term outcome. Current data from the Swedish Knee Arthroplasty Register annual report from 2007 show that for patella implants performed since 1996, non-resurfacing is associated with a 1.3-times higher cumulative revision rate than resurfacing in the setting of osteoarthritis, and 1.9-times higher for rheumatoid arthritis. The authors suggested that this is directly related to the need for secondary patella resurfacing because of patellofemoral pain (Robertsson and Lidgren 2007). These promising results suggest that the mechanical forces of the patella prosthesis may not affect tibial or femoral components in the medium to long term, and they present an ideal opportunity for follow-up in the future.

Osteoarthritis is currently the major reason for total knee arthroplasty performed in Australia, constituting 97% of initial diagnoses. We found that the 5-year CPR was lower in the R group in the setting of both osteoarthritis and other diagnoses. These values in the setting of other diagnoses (such as rheumatoid arthritis) support data from the Swedish Knee Arthroplasty Register (Robertsson and Lidgren 2007); however, generally speaking the published literature has yet to show a difference in outcome for resurfaced patellas in terms of revision rates (Shoji et al. 1989, Kajino et al. 1997, Moran and Horton 2000, Gioe et al. 2007). Potentially confounding factors such as age, gender, and mean time to revision had no effect on our results.

Our study defined both “patella only” and “insert and patella” as patella additions, and this took account of surgeons who may have routinely changed the insert at revision. Revision procedures for “insert only” were not part of our analysis. Our data suggest that there was no difference in the revision rate between “insert and patella” revisions and “patella only” revisions, and both “‘insert and patella” and “patella only” in the NR group had a higher revision rate than in the R group (p < 0.001 and p < 0.001, respectively; data not shown).
